# Application of intraoperative infrared thermography in bypass surgery for adult moyamoya syndrome: A preliminary study

**DOI:** 10.3389/fneur.2023.1174072

**Published:** 2023-03-30

**Authors:** Jinghui Lin, Yiwen Wu, Xinpeng Deng, Shengjun Zhou, Yuchun Liu, Junjun Zhang, Yiyong Zeng, Xianru Li, Xiang Gao, Bin Xu, Chenhui Zhou

**Affiliations:** ^1^Department of Neurosurgery, Ningbo First Hospital, Ningbo University, Ningbo, Zhejiang, China; ^2^Department of Neurosurgery, Huashan Hospital, Fudan University, Shanghai, China

**Keywords:** moyamoya syndrome, infrared thermography, indocyanine green fluorescein videoangiography, cerebral revascularization, moyamoya disease

## Abstract

**Background and objectives:**

Cerebral revascularization surgery is the mainstay of treatment for moyamoya syndrome (MMS) today, and intraoperative determination of the patency of the revascularized vessel is a critical factor in the success of the procedure. Currently, major imaging modalities include intraoperative indocyanine green (ICG) videoangiography (ICG-VA), digital subtraction angiography (DSA), and vascular ultrasound Doppler. Infrared thermography is a modern imaging modality with non-contact devices for the acquisition and analysis of thermal data. We aimed to investigate the feasibility and advantages of infrared thermography in determining anastomotic patency during MMS surgery.

**Methods:**

Indocyanine green videoangiography and infrared thermography were performed simultaneously in 21 patients with MMS who underwent bypass surgery. The detection result of vessel patency was compared, and the feasibility and advantages of infrared thermography were assessed.

**Results:**

The patency of the anastomosis was accurately determined in 21 patients using either ICG angiography or infrared thermography. In 20 patients, the results of infrared thermography showed that the vascular anastomosis was unobstructed, and there was an agreement with the subsequent results of ICG-VA. In one patient, we suspected inadequate patency after testing the anastomosis with infrared thermography, and the results of ICG-VA evaluation of the anastomosis confirmed that there was indeed an anastomotic obstruction.

**Conclusion:**

Compared with ICG-VA, infrared thermography might offer an alternative non-invasive, contrast-free option in assessing anastomosis patency compared with ICG-VA, and it is likely to become more widely used in the clinic in the near future.

## 1. Introduction

Moyamoya syndrome (MMS) is a cerebrovascular disease characterized by progressive stenosis or occlusion of the bilateral internal carotid, middle cerebral, and anterior cerebral arteries at their origins, resulting in the formation of an abnormal vascular network at the base of the skull (1). The disease earns its name as the abnormal vascular network appears as a “puff of smoke” (“*moyamoya*” in Japanese) in cerebral angiographic images, while the pathogenesis of MMS remained unclear today. MMS is highly prevalent in East Asian countries including China, Japan, and Korea. The main clinical manifestations of MMS include cerebral hemorrhage and ischemia. Surgical revascularization is now the mainstay of treatment, including direct, indirect, and combined bypass. Surgical techniques in cerebral revascularization have developed at a rapid pace to increase cerebral blood flow and reduce the risk of stroke. Nevertheless, there are increasing reports regarding the complications following revascularization surgery, leading to prolonged hospitalization stay and some of which are irreversible (2, 3). Currently, the success of bypass surgery continues to be evaluated by the degree of anastomotic patency rather than the incidence of postoperative complications. Yet, all current methods of intraoperative visualization of vascular structures have limitations and drawbacks (4).

Indocyanine green videoangiography is now widely performed (5). The patency of the anastomosis is assessed by intravenous ICG injection (6). However, contrast agents sometimes lead to allergic reactions and it requires a long time for contrast agent metabolism. Therefore, there has been a search for a way to both directly observe blood flow and assess anastomotic patency without using intravenous contrast agents. Infrared thermography involves temperature measurement of the infrared radiation received from the tissue surface and visualization of the data to compare temperature differences within the region of interest. Nowadays, infrared thermography has been widely used in clinical applications, and studies have shown good results in the prediction of flap graft survival, assessment of blood flow reconstruction in the limb, and evaluation of postoperative area infection (7, 8). We attempted to apply infrared thermography to determine anastomotic patency after cerebral blood flow reconstruction to provide a new means of intraoperative monitoring of MMS.

## 2. Methods

### 2.1. Patients

This continuously enrollment research enrolled 21 patients diagnosed as MMS qualified for surgery and admitted from October 2021 to January 2022, all of whom underwent superficial temporal arterycomputed tomography to middle cerebral artery (STA-MCA) bypass surgery. The study protocol was approved by the local institutional review board, and the experiment was conducted by the relevant institutional guidelines and in compliance with the Declaration of Helsinki as revised in 1983. The diagnosis was based on the diagnostic criteria for MMS proposed by the Committee on the Pathology and Treatment of Spontaneous Occlusion of the Circle of Willis in 2012 (9). Written informed consent was obtained from all patients.

### 2.2. Surgical procedure

All of the surgeries were performed by the same experienced neurosurgeon. After induction of general anesthesia, the patient was placed in the supine position with the head tilted 60° to the side. After sterilization and draping, a 15-cm arc-shaped incision on the frontotemporal scalp was made, cutting through the temporalis muscle. The temporalis muscle flap was then folded anteriorly, and care had been taken to protect the STA. A 9 cm × 8 cm craniectomy was created without damaging the middle meningeal artery. It was visible at this moment that the dura mater was in moderately high tension, with no abnormality in the color and pulsation of the brain. Next, the dura was lifted, and the STA was dissected from the surrounding tissue at the parietal branch. With the radial incision of the dura performed, the thin arteries over the surface of the brain were visible, and the recipient blood vessel in the bypass region was subjected to surgery (STA-MCA), after which the patency of the vascular anastomosis was assessed by the simultaneous application of ICG and infrared thermography. Routine computed tomography scans were performed postoperatively to seek out any secondary postoperative infarction. We did not perform routine postoperative angiography, and it was done at follow-up at 3 months.

### 2.3. Intraoperative infrared thermography

**Figure 1:**
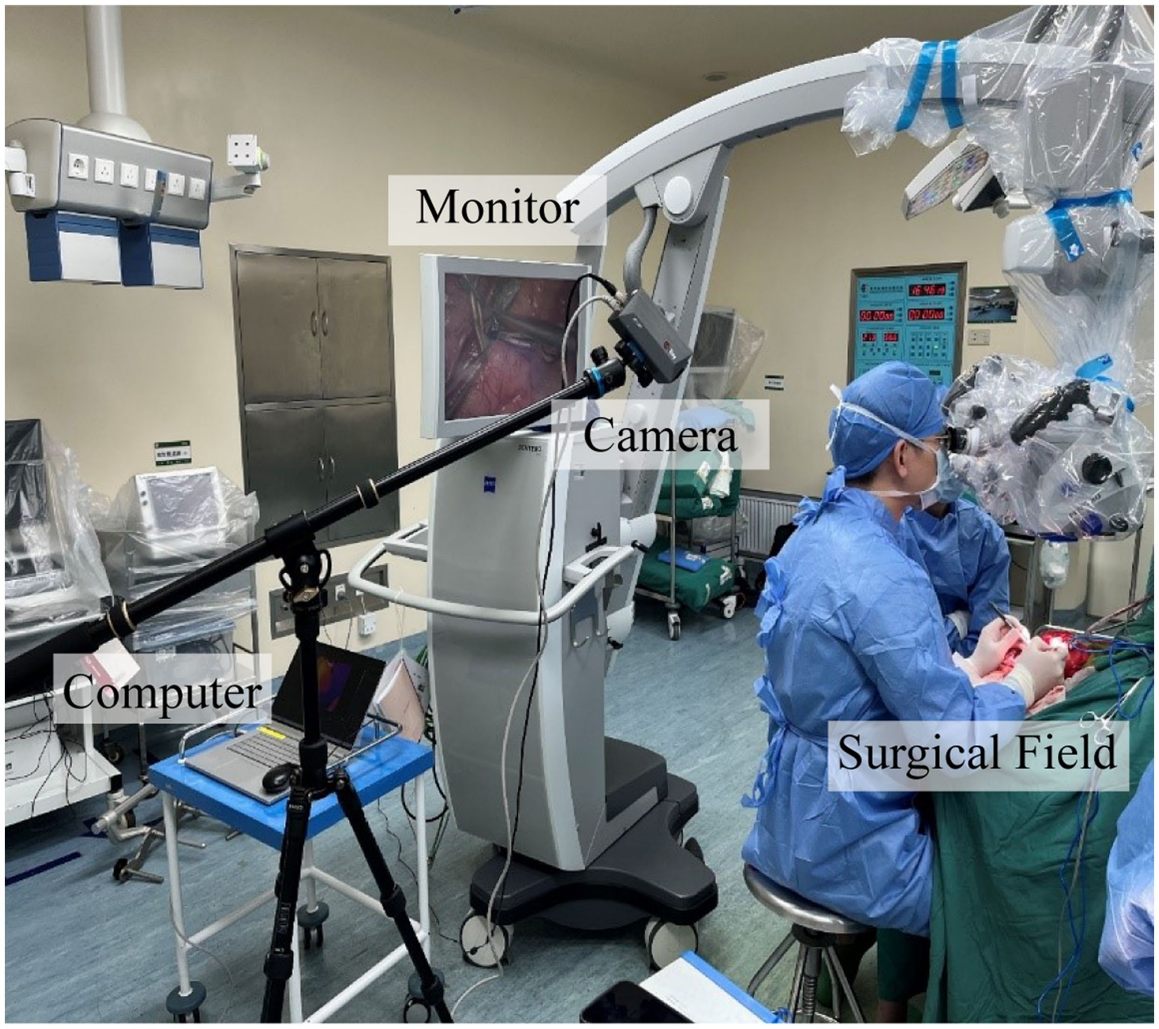
Photograph of the infrared imaging system in the operating room.

For our research, we used an AT1280 digital infrared camera (Electronically Modulated Online Thermometry, Iray Technology Co., Ltd., China), which was used to image local temperature gradients across the cerebral cortex by passively detecting infrared emissions. We kept the room temperature at 23°C to maintain the background temperature of the cortical surface as much as possible. After completion of the anastomosis, the high-resolution infrared camera of the infrared imaging system was set 500 mm above the brain obliquely to continuously monitor blood flow for up to 3 min, including the time to place and release the vascular blocking clips (Figure 1).

The infrared imaging system consisted of three parts: an infrared camera, the main computer, and a monitor. The thermal imaging camera was 70 mm × 63 mm × 143 mm and was set up on a collapsible tripod and connected to the main computer *via* a network cable for imaging analysis. The camera used a vanadium oxide uncooled infrared focal plane detector with a 12 μm pixel pitch, 1,280 × 1,024 pixels, a 19 mm lens, and an instantaneous field of view of 0.63 mrad. The camera can be set up at 0.5 m from the cerebral cortex to obtain an infrared field of view of 400 mm × 320 mm and can observe a minimum target diameter of 0.315 mm. The detectable wavelength band was 8–14 μm. No contrast agent or radiation was used to obtain the image. The recording speed was 15 frames per second. All images were stored on the installed computer and recorded with digital video equipment. The assessment of the patency of the vascular anastomosis can be performed directly on the monitor.

### 2.4. Intraoperative indocyanine green videoangiography

Indocyanine green videoangiography was performed with an operating microscope (Opmi Pentero 900, Carl Zeiss) equipped with a fluorescent light source (wavelength 700–850 nm) and an infrared-sensitive camera. The microscope was placed perpendicular to the study area at a distance of approximately 300 mm. During the ICG-VA, the room lights are dimmed and a weight-adapted dose of 0.25 mg/kg of ICG dissolved in 10 ml of saline is injected *via* a central venous catheter. Once the ICG reaches the corresponding area, it receives excitation from near-infrared light and emits fluorescence, which is captured and recorded by the camera equipment. The operator assesses the patency of the vascular anastomosis by observing the fluorescence image microscopically. In all cases, intraoperative infrared thermography is performed before ICG-VA.

### 2.5. Acquisition of data

Patient data about pre-and post-operative radiological images are obtained from the department’s digital patient management software. Intraoperative findings are assessed by video analysis and analysis of operative reports.

## 3. Results

### 3.1. Demographics

A total of 21 patients with MMS who underwent STA-MCA anastomoses surgery were included. Infrared thermography and ICG-VA were both applied to assess the anastomotic patency in the 21 patients. The patient’s ages ranged from 27 to 59 years (median 46 years). The 17 of our patients presented with ischemic stroke symptoms, such as hemiparesis. Four patients had a history of cerebral hemorrhage (Table 1).

**Table 1:** Clinical characteristics of the study population.

Case no.	Sex	Age	Symptoms	Operated side
1	Male	54	Recurrent stroke/TIA	Right
2	Female	56	Hemiparesis	Left
3	Male	57	Recurrent stroke/TIA	Right
4	Female	47	Recurrent stroke/TIA	Right
5	Male	30	Recurrent stroke/TIA	Right
6	Female	54	Recurrent stroke/TIA	Right
7	Male	34	Hemorrhage	Left
8	Male	27	Recurrent stroke/TIA	Left
9	Female	58	Recurrent stroke/TIA	Right
10	Male	56	Recurrent stroke/TIA	Left
11	Male	37	Hemiparesis	Left
12	Male	32	Recurrent stroke/TIA	Left
13	Male	36	Hemorrhage	Right
14	Female	32	Hemorrhage	Right
15	Female	54	Recurrent stroke/TIA	Right
16	Female	54	Recurrent stroke/TIA	Right
17	Female	34	Hemorrhage	Right
18	Female	59	Recurrent stroke/TIA	Right
19	Male	39	Recurrent stroke/TIA	Left
20	Female	58	Hemiparesis	Left
21	Male	57	Recurrent stroke/TIA	Right

### 3.2. Assessment of anastomotic patency

**Figure 2:**
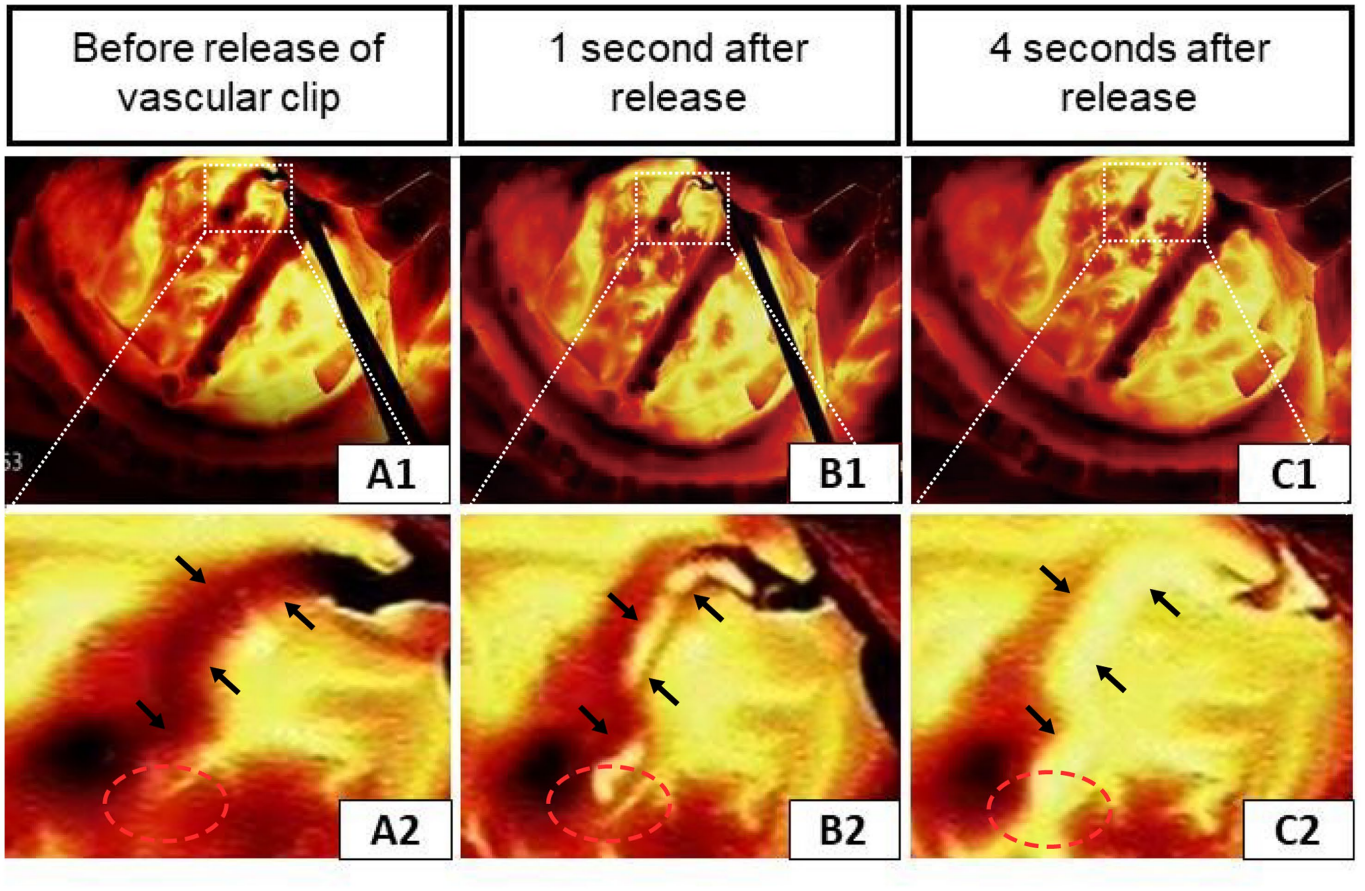
Infrared thermography of a case during right STA-middle cerebral artery (M4) bypass surgery. **(A1–C1)** Infrared thermographic images of the vessel clip before and shortly after release. **(A2–C2)** Enlarged views correspond to the upper (the black arrows indicate the STA and the red circles show the anastomosis of the donor and recipient vessels).

Figure 2 illustrated an infrared thermographic image obtained during a right STA-MCA (M4) bypass surgery in one case. Before the release of the vascular blocking clip, the temperature of the anastomosis and the donor vessel (STA) was low and almost no blood flowed through the anastomosis, corresponding to the dark area in Figures 2A1, 2. After releasing the vascular clip from the STA, it was clear in Figures 2B1, 2 that blood with a lower temperature relative to the cerebral cortex flows from the donor’s vessel through the anastomosis to the recipient’s vessel, indicating good patency of the anastomosis. Furthermore, the distal branch vessels appeared for a split second in the thermal imaging as a dark image, indicating a positive flow of blood from the donor’s vessels toward the cerebral cortex. Figures 2C1, 2 showed the situation at 4 s after the release of the vascular clip. All vessels became highlighted in color. These findings demonstrated the success of the revascularization and show that infrared thermography can make a correct assessment of anastomotic patency.

### 3.3. Infrared thermography versus ICG fluorescence imaging

**Figure 3:**
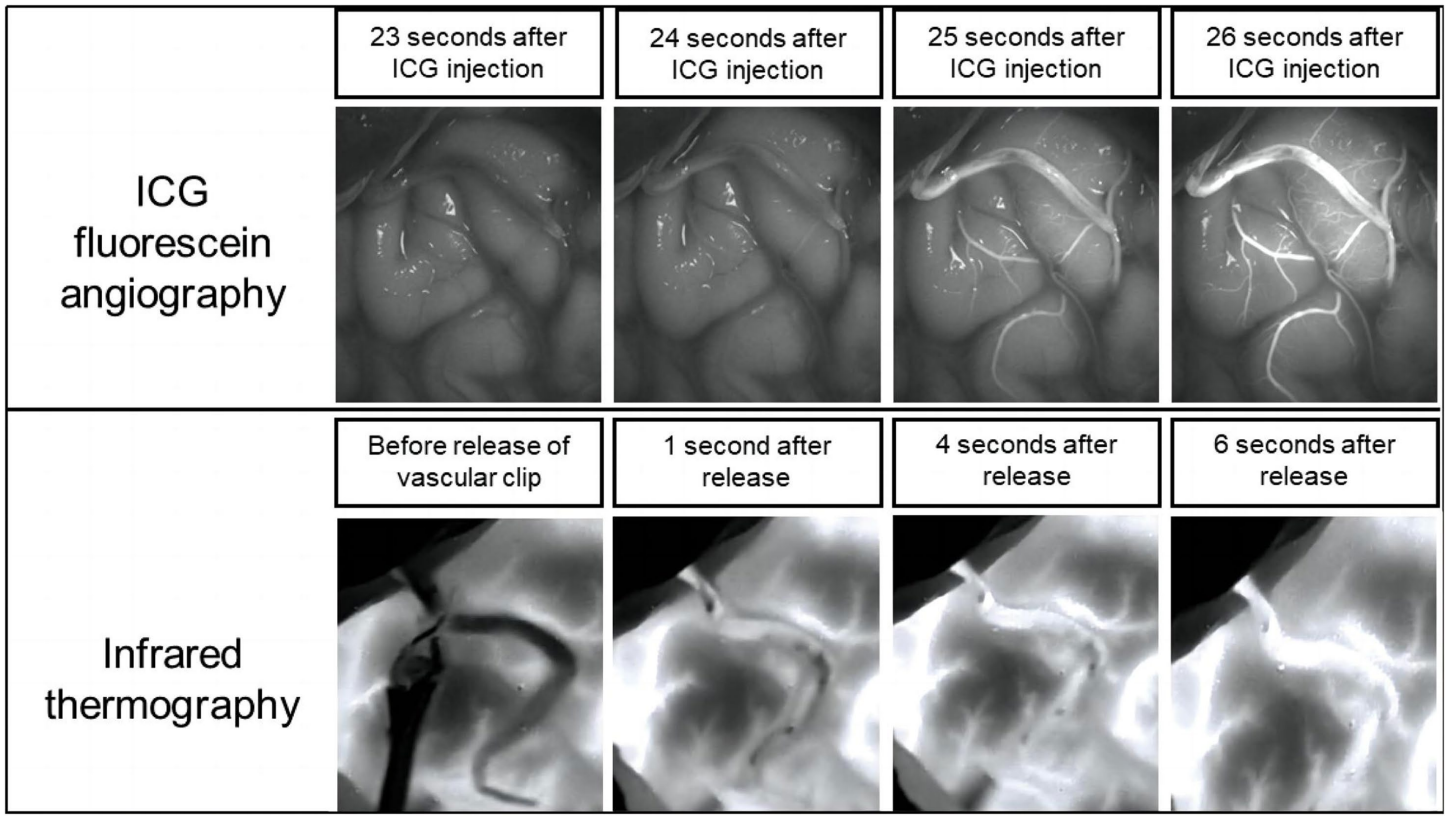
Comparison of ICG-VA and infrared thermography.

We carried out infrared thermography followed by ICG-VA in each MMS patient. The validity of the infrared thermography technique was confirmed by comparing the results with the ICG assessment. Figure 3 shows that the ICG was visualized after ICG injection, unambiguously confirming the patency of the anastomotic vessels, and the same results were visualized on the infrared thermography of the same patient. In our study, a total of 21 patients were treated the same way. In 20 patients, the results of infrared thermography showed that the vascular anastomosis was unobstructed, and there was an agreement with the subsequent results of ICG-VA.

**Figure 4:**
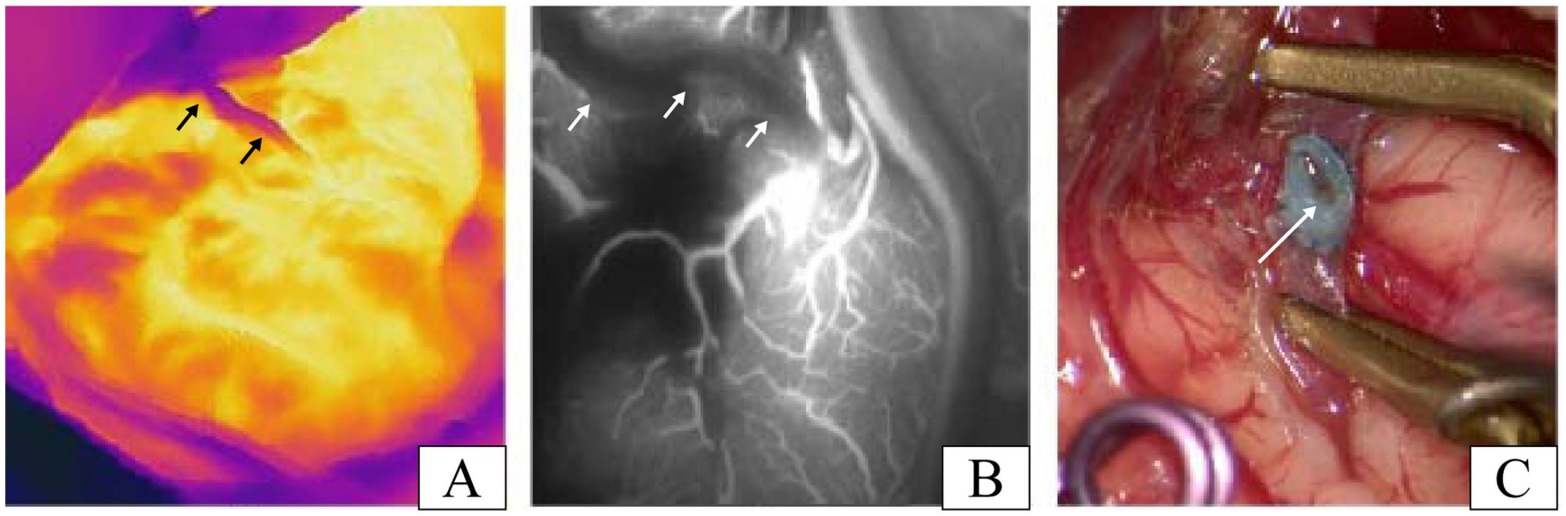
A patient with inadequate patency of vascular anastomosis in our study. **(A)** Infrared thermographic results of this patient after the release of the vascular clip (the black arrows denote the STA). **(B)** Image results of the ICG-VA after the release of the vascular clip in this patient (the white arrows indicate the STA). **(C)** Thrombosis was found after reopening the recipient artery and was the cause of the insufficient patency of the vascular anastomosis in this case (the white arrow shows the thrombosis in anastomosis).

Unexpectedly, in one patient, we suspected inadequate patency after testing the anastomosis with infrared thermography, and the results of ICG-VA evaluation of the anastomosis confirmed that there was indeed an anastomotic obstruction (Figures 4A,B). After opening the anastomosis, it is obvious from Figure 4C that intravascular thrombosis was the exact cause of the anastomotic opacity in this case. This further proved the surprisingly consistent results of infrared thermography and ICG-VA in assessing anastomotic patency. The case was subsequently re-anastomosed by removing the anastomotic thrombus. Three patients developed transient neurological deterioration after surgery, including aphasia in two cases and contralateral limb asthenia in one case. Patients with these symptoms gradually resolved within 5–7 days postoperatively, and none of them experienced permanent neurological deterioration. All patients had a disappearance or improvement of transient cerebral ischemic symptoms during the follow-up period.

## 4. Discussion

Rapid and accurate assessment of anastomotic vessel patency has been a crucial aspect of cerebrovascular surgery due to the extreme difficulty of vascular anastomosis and the lack of an objective basis for determining patency by visual inspection. In this study, we demonstrated the usefulness of infrared thermography in identifying anastomotic vessel patency during the revascularization process of patients with MMS. Surprisingly, our results demonstrated that infrared thermography is equally effective in determining anastomotic vessel patency compared to ICG-VA.

Infrared thermography in current clinical practice tends to focus on the assessment of flap blood supply for tissue defect repair (10, 11). Apart from traditional applications, we propose for the first time the application of infrared thermography to assess anastomotic vascular patency during direct revascularization procedures for the treatment of MMS. Upon completion of the vascular anastomosis, the blood flow in the clamped donor and recipient arteries is stationary, thus its temperature is almost similar to room temperature. Immediately after opening the vascular blocking clamp, the hemodynamic behavior of the donor and recipient vessels changes, so that the flowing blood rapidly returns to the same temperature as the human body. The dynamic distribution of blood flow at different temperatures resulting in significant temperature contrasts between the vessels at different sites and between the surrounding tissues. The temperature contrast results in different intensities of infrared radiation being acquired by the camera and converted into a pseudo-color thermographic video/image, highlighting areas of higher temperature with highlighting colors, along with darker areas of lower temperature. The higher the temperature, the brighter the area. The technical advantage of infrared thermography is that the pseudo-color video/images allow the surgeon to understand the patency and hemodynamic behavior of the donor and recipient vessels to make an effective and accurate intraoperative assessment of anastomotic patency.

Numerous vascular imaging techniques to date were developed. DSA is considered the standard for the diagnosis and evaluation of moyamoya disease and moyamoya syndrome (12). However, DSA is an invasive procedure with a risk of inguinal hematoma and transient neurological symptoms (13). Besides, the DSA procedure is complex and expensive to perform, requires bulky supporting instruments and specific operating rooms, and exposes both patients and operating surgeons to radiation, all of which makes it inadequate for determining the patency of anastomosed vessels. In contrast to DSA, infrared thermography is based on the blood flow to visualize changes in temperature modulation to show flow and assess patency. It is an unparalleled advantage in terms of both ease of operation and cost-effectiveness.

Microvascular ultrasound Doppler can assess anastomotic patency by quantifying vascular hemodynamic data (14). But the limitations come with it. Firstly, the ultrasound Doppler technique places too much emphasis on the probe-vessel angle, and measurements at different angles will give variable results, making the results too disparate. Secondly, microvascular ultrasound Doppler does not visualize the morphology of the vessel and is even less sensitive to tiny vessels (15, 16). Infrared thermography solves the problems of inaccurate measurement and indirect observation of blood vessels with the visualization of blood flow.

Indocyanine green videoangiography is currently the most commonly used and standard method for intraoperative assessment of anastomotic patency, with excellent temporal and spatial resolution, and is easy and simple to perform. However, it is of great importance to note that a few patients are allergic to ICG dye. Although the incidence is very rare, this is not a risk-free procedure. It is worth mentioning that the ICG-VA must be kept clean in the surgical field of view during the angiography procedure and that the viewing angle of the microscope has a major impact on the imaging results, requiring some experience of the operators in assessing anastomotic stability. If the anastomosis is suspected to be obstructed, it needs to be adjusted or re-sutured and re-imaged. Studies have shown that it takes at least 15 min for the ICG to be completely metabolized in the previous residual vessel, which undoubtedly increases the time cost (17). Compared to ICG fluorescein imaging, infrared thermography enhances the safety of the procedure without requiring contrast agents, clearly shows the blood flow into the anastomosis, and can be used at any time, repeatedly and multiple times.

## 5. Limitations

Admittedly, infrared thermography is far from perfect technology. One of the limitations of our study is that no corresponding software development applications have yet been seen, making infrared thermography currently only able to show the direction of blood flow and not yet able to assess the distribution of blood flow and perform the corresponding quantitative analysis. However, as technology develops, exploration and research related to infrared thermography in the cerebrovascular field would facilitate techniques to help us overcome this limitation. Furthermore, our study was a single-center and retrospective study, which resulted in limited persuasiveness and restricted generalizability. To further validate our findings, our next step is to conduct a multi-center clinical trial with a large sample-size.

## 6. Conclusion

Our study confirms that in vascular anastomosis procedures for MMS, infrared thermography can achieve the same assessment effect as ICG and is safe and easy to use, allowing direct, dynamic, real-time visualization of cerebral blood flow and providing neurosurgeons with new ideas for assessing anastomotic patency, and it is likely to become more widely used in the clinic in the near future.

## Data availability statement

The raw data supporting the conclusions of this article will be made available by the authors, without undue reservation.

## Ethics statement

The studies involving human participants were reviewed and approved by ethics committee of Ningbo First Hospital. The patients/participants provided their written informed consent to participate in this study. Written informed consent was obtained from the individual(s) for the publication of any identifiable images or data included in this article.

## Author contributions

XG and BX: conceptualization and project administration. SZ and YL: methodology. XD: software and investigation. JZ and YZ: validation. XL: formal analysis. JL and YW: writing—original draft preparation. CZ: writing—review and editing and funding acquisition. XG: supervision. All authors contributed to the article and approved the submitted version.

## Funding

This research was funded by grants from the Ningbo Health Branding Subject Fund (PPXK2018-04), Ningbo Science and Technology Innovation 2025 Major Project (2022Z134), and Ningbo Top Medical and Health Research Program (2022020304) to XG, National Natural Science Foundation of China (82101354) to CZ and Zhejiang Traditional Chinese Medicine Scientific Research Fund Project (2021ZA129) to JL.

## Conflict of interest

The authors declare that the research was conducted in the absence of any commercial or financial relationships that could be construed as a potential conflict of interest.

## Publisher’s note

All claims expressed in this article are solely those of the authors and do not necessarily represent those of their affiliated organizations, or those of the publisher, the editors and the reviewers. Any product that may be evaluated in this article, or claim that may be made by its manufacturer, is not guaranteed or endorsed by the publisher.
